# Psychological Resilience Interventions for Adolescents during the COVID-19 Pandemic

**DOI:** 10.3390/bs13070543

**Published:** 2023-06-29

**Authors:** Jingwen Xing, Xiaofeng Xu, Xing Li, Qing Luo

**Affiliations:** 1 School of Primary Education, Shanghai Normal University Tianhua College, Shanghai 201815, China; wxing@chapman.edu; 2 School of Health, Shanghai Normal University Tianhua College, Shanghai 201815, China; xxf1132@sthu.edu.cn; 3 School of Psychology, Central China Normal University, Wuhan 430079, China; 4 School of Public Policy and Administration, Nanchang University, Nanchang 330031, China

**Keywords:** COVID-19, adolescents, resilience, resilience intervention

## Abstract

The COVID-19 pandemic has had severe mental health effects on adolescents. Psychological resilience is the ability to recover quickly from adversity and can help adolescents cope with the stress and dangers brought by the pandemic better. Therefore, the current study aimed to explore the developmental pattern of psychological resilience in adolescents and to find the sensitive period for psychological resilience intervention to promote resilience in adolescents during the pandemic. The study measured the psychological resilience of a total of 559 adolescents using the Connor-Davidson resilience scale (CD-RISC) in four grades: grade 7 and grade 8 in a junior high school, and grade 10 and grade 11 in a high school. It was found that the resilience level of the adolescents decreased in grade 10 and then increased significantly in grade 11 (*F* = 4.22, *p* = 0.006). A 4-week resilience intervention was conducted in the four grades using both psychological course training and physical training. The results revealed that the psychological course training was effective in promoting resilience in the 7th (*F* = 4.79, *p* = 0.03) and 8th (*F* = 4.75, *p* = 0.03) grades, but not in the 10th and 11th grades. The result suggests that the 7th and 8th grades may be a critical period for psychological resilience interventions for adolescents.

## 1. Introduction

Since its outbreak in 2019, COVID-19 has caused more than 600 million infections and killed more than 6 million people worldwide [1]. Despite the currently reduced mortality of COVID-19, the recovered survivors still suffer physical consequences, including direct brain damage, having an impact on mental health [2,3,4,5]. Even among the healthy public and individuals who have not experienced physical harm from COVID-19, the mental health effects brought by COVID-19 and its control measures should not be underestimated. Negative effects of COVID-19 on mental health in the population at large have been found across the world and have even caused mental disorders for some people [6,7,8,9,10]. Our study focuses on implementing resilience intervention for adolescents who have also been affected by the pandemic [11,12,13,14,15], and aims to help them better navigate this process.

Among the underage groups, adolescents are most negatively affected by the pandemic [7,12]. Adolescence is often referred to a unique stage called “the dangerous period” [16]. During adolescence, there is an accelerated process of cognitive development and identity formation, an increase in impulsive needs and emotional intensity, and the onset of individualization. Therefore, individuals may experience specific challenges and conflicts during this period [17]. Adolescence is prone to various mental and physical developmental vulnerabilities and dysfunctions, such as mood disorders, substance abuse, and obesity [18]. In addition to “growing pains”, teenagers will also encounter many troubles, such as academic pressure and relationship problems (romantic, parent–child, and interpersonal) [19]. The pandemic seemed to press the accelerator button on the fluctuations of “stormy” adolescence [7,11]. Youths show more depressive symptoms, anxiety symptoms, and other negative emotions from before to during the pandemic [15,16,17]. Among the underage groups, teenagers also experience the highest prevalence and severity of mental health problems compared to younger groups [20]. Schmidt and his colleagues compared the effects of the COVID-19 pandemic on mental health in three age groups (1–6 years, 7–10 years, and 11–19 years), with adolescents reporting the greatest increase in emotional problems [21]. Similar studies have shown gradual and substantial increases in depression, anxiety, and stress in adolescents [14]. More than one fifth of youngsters experienced adverse effects on their mental health during the COVID-19 pandemic in China [11]. COVID-19 may interfere with pubertal developmental tasks and constitute a “perfect storm” of difficulties for adolescents [16]. Furthermore, the epidemic has brought changes and pressure to people’s lives, such as the financial situation of parents, which not only directly affects the living standards of teenagers, but also hurts the parent–child relationship [22,23].

Fortunately, several world health agencies including WHO, UNICEF, and many others have issued intervention manuals to help parents protect the mental health of their children during COVID-19 [24]. However, these guidelines are primarily aimed at calming the child down and maintaining daily routines [25]. As a way to mitigate the negative impact of COVID-19 on youths, psychological resilience may play an essential role, as it refers to effective coping and adaptation in the face of loss, difficulty, or adversity [11]. The relationship between COVID-19 and stressful experiences could be mediated by resilience [11]. Numerous studies have found a significant influence of resilience on adolescent mental health during the pandemic. There was a significant negative correlation between resilience and sense of danger and distress symptoms [11]. Less resilient teenagers were significantly more likely to have poor mental health outcomes, including more severe symptoms of depression and anxiety, and even more severe suicidal ideation [7,26]. In contrast, a high level of resilience provides protection against various mental health conditions [19], as it can significantly predict decreased severity of anxiety and depression during the pandemic [11], and is also associated with decreased psychological distress and increased positive experiences [27]. More resilient adolescents display more prosocial attitudes and fewer mental health problems [28]. Developing resilience-building interventions for youngsters in this age group to efficiently promote mental health recovery during pandemics is becoming a consensus [11,27], as targeted psychological resilience training is found to improve their mental health and enhance their ability to cope with the pressure brought by COVID-19.

Adolescence may be a critical period for developing resilience-related skills at the same time [29,30]. On the one hand, adolescence is a “leap period” of physical and mental development, especially the critical period for the maturation of the neurophysiological development process, which underlies higher cognitive functions and social and emotional behavior [9,10,12,13]. During this period, the human brain undergoes a substantial structural and functional reorganization [31]. Brain regions and systems that support resilience and related skills, such as the prefrontal cortex [32], amygdala, striatum [33,34,35], dopamine system [36], and hippocampus [37] all develop obviously and mature continuously during adolescence. The developmental period is also characterized by increased plasticity, during which neurocognitive networks evolve and are shaped through reciprocal interactions between the brain, body, behaviour, and the environment [38]. The brain is particularly susceptible as well as to experience and intervention [36]. Adolescence may give a unique opportunity to nurture resilience and provide a “recalibration” window that promotes intervention [37]. Intervention in adolescence may be exceptionally responsive and more productive than in other periods [38].

On the other hand, the pandemic itself has brought new challenges to the already dangerous period of adolescence. Adolescents have been affected by the limitations brought on by the pandemic, such as school closures and the shift to online learning from home. They have experienced less physical activity, irregular or inadequate sleep, reduced in-person peer communication and peer support, and have spent more time online [21,39,40]. In addition, a series of economic or productivity changes brought about by the pandemic are more likely to cause distress in parent–child interactions and have a negative impact on their relationship [39,41]. In addition, isolation policies during the pandemic have increased loneliness among young people, making them more vulnerable to mental health problems, further exacerbating the negative impact of COVID-19 on them [42,43].

Adverse events have an impact on youths in terms of brain structure and function, emotional regulation, and cognitive function [17]. Despite the strong association between stressors and mental health, not all adolescents exposed to adversity develop psychiatric disorders [44]. The developing brain, although more vulnerable to adversity, also offers more opportunities for positive change through intervention [45]. Psychological resilience can aid adolescents in coping with problems and dangers [16]. Interventions at this time are essential for individuals to cope with difficulties caused by developmental processes or pandemics, as well as a combination of both.

Psychological resilience interventions for teenagers can alleviate stress reactions, increase their well-being and mental health, and promote their long-term ability to overcome adversity [45,46,47,48,49]. The majority of junior high school students (i.e., grade 7, grade 8, and grade 9) are in early adolescence, and the majority of high secondary students (i.e., grade 10, grade 11, and grade 12) are in mid to late adolescence. There may be significant variations in the developmental status of secondary school students at different grades since individuals develop rapidly in adolescence. For example, during childhood and adolescence, cortical white matter volume continues to increase while cortical gray matter volume simultaneously decreases [50]. Developmental improvements in response inhibition, error monitoring, task switching, and probability updating typically last into early adolescence, while developmental enhancements in working memory manipulation, feedback learning, delay discounting, and emotion regulation are observed throughout adolescence and into early adulthood [51,52,53]. The hippocampus, which promotes resilience through the narrative of emotional responses, also develops during adolescence [36,54,55].

By carrying out investigations and interventions, our study aims to answer the following questions:

1. Are there differences in the levels of psychological resilience of junior high and high school students in different grades, and if so, how do these variations manifest?
2. Does the resilience intervention for junior high and high school students have different effects due to their different development status?
3. In which grades do interventions produce the best results?

We surveyed the resilience levels across four grades to assess the developmental pattern of adolescents and employed both psychological course training and physical training, which are now widely used for resilience training to find the most efficient period of resilience intervention by comparison [45,48]. Considering that adolescents undergo continuous development and maturation throughout adolescence, we hypothesized that (1) their mental resilience should also increase with each grade. At the same time, (2) the intervention may be more effective for adolescents in lower grades because teens in higher grades are in late adolescence, closer to adulthood, and may exhibit reduced susceptibility to external influences.

## 2. Materials and Methods

### 2.1. Participants

The intervention randomly selected participants from two secondary schools in Wuhan in 2022. As the epicenter of the COVID-19 outbreak, Wuhan may be the site where adolescents could have experienced the greatest need for resilience with respect to COVID-19 [54]. A total of 559 participants (N = 559) were ultimately recruited, covering two grades in a junior high school (grade 7 and grade 8) and two grades in a high school (grade 10 and grade 11). The mean age of participants was 14.19 (*SD* = 1.51) years, ranging from 12 to 17 years. Boy and girl participants constituted 49.73 and 50.27% of the sample, respectively. This study was approved by the Ethical Committee for Scientific Research at the researchers’ affiliated institution, and complied with the ethical guidelines protecting human participants. All participants participated in the experiment voluntarily, signed written informed consent, and received a small gift as an incentive.

All the data came from participant self-reporting; we changed the guidance and scoring to avoid common method bias during data collection [55]. We also conducted Harman’s single-factor test by loading all variables into an unrotated exploratory factor analysis [51]. The first factor explained the variance by 30.79%, less than the 40% required by the critical standard. These results suggest that common method bias is not a major concern in this study.

### 2.2. Procedure

A cluster randomized control trial was conducted with classes as the unit of randomization. Three classes from each of the four grades were randomly selected to participate in the study and randomly allocated to either the control group (each grade recruited one class into the control group) or the intervention group (each grade recruited two classes and was assigned to different intervention groups). Every participant completed the pre-intervention scale (baseline) before the intervention, which is used to assess the adolescent’s resilience levels [56,57]. Then, there was a four-week intervention for each intervention group, but no intervention for the control group during this period. After the intervention session, every participant completed the same scale as before, which was used to assess the impact of the intervention [56,57]. The investigator conducted pre and post tests and monitored the entire intervention process.

#### 2.2.1. Intervention Group

##### Psychological Course Training

The psychological course training protocol was based on the three factors (i.e., tenacity, strength, and optimism) of the Connor-Davidson resilience scale (Table 1) which is also appropriate for investigating resilience during COVID-19 [58,59]. There were four classes from four different grades. The psychological course was conducted at the same time respectively.

##### Physical Training

The hand-muscle developer was adopted as the tool for physical training [60,61]. Before the start of the training session, the experimenter informed participants of the safety concerns, after which the participants followed the instructions to grip the hand-muscle developer and record the initial level of maximum grip strength they could achieve. At the start of the session, the participants zeroed out their hand-muscle developer count and completed a time-limited grip challenge.

There were two rounds of the grip challenge. The first round of the grip challenge was set at 10 kg for boys and 5 kg for girls. Participants were asked to press the hand-muscle developer as many times as they could in 1 min, then recorded the number of presses they actually made in 1 min. The second round of the grip challenge was played with the other hand, and was set at 20 kg for the boys and 10 kg for the girls. Participants were required to press the hand-muscle developer as many times as they could in 1 min and recorded the number of presses they actually made again.

There was a three-minute break between the two rounds of the challenge. The experimenter encouraged students to persevere in their tasks and also encouraged them to cheer each other on and keep challenging themselves. There were four classes from four different grades that conducted the physical training at the same time, respectively.

#### 2.2.2. Control Group

There was no intervention from the research team to the control group. They completed the pre-intervention scale (baseline) and post-intervention scale at the same time as in the intervention group only.

### 2.3. Measures

The Connor-Davidson resilience scale (CD-RISC) was used to assess resilience levels [56]. The Chinese version was developed by Yu and Zhang [57] with good internal consistency (Cronbach’s *α* = 0.91) and test–retest reliability (intraclass correlation coefficient = 0.87). There are 25 items that belong to three factors: tenacity, strength, and optimism. The factor tenacity describes an individual’s equanimity, promptness, perseverance, and sense of control when facing situations of hardship and challenge. Strength focuses on the individual’s capacity of recovering and becoming strong after setbacks and past experiences. Optimism reflects the individual’s tendency to look on the positive sides of things and trusting one’s personal and social resources. Participants were asked to respond on a 5-point Likert scale, from 1 (not true at all) to 5 (true all the time). The total score of the scale represents the overall resilience level, and the scores of the corresponding questions constitute the scores for each of the three factors in each subscale. A higher score indicates a higher level of resilience/tenacity/strength/optimism.

### 2.4. Statistical Analyses

To discover the developmental pattern of adolescent resilience, one-way ANOVA, which is a widely used method [62,63], was used to compare the resilience score and three factors (tenacity, strength, and optimism) across all grades. To compare the effect of psychological resilience intervention on adolescents of different grades, 3 (each group: psychological course training, physical training, control group) × 2 (time: before and after intervention) repeated measures ANOVA was used to examine the intervention effect in each grade.

## 3. Results

### 3.1. The Resilience Development Pattern in Adolescents

The resilience score significantly differed across grades, *F*(3,555) = 4.22, *p* = 0.006 < 0.01, η^2^*_p_* = 0.02; the resilience score in 10th grade (*M* = 53.69, *SD* = 11.53) was significantly lower than in 11th grade (*M* = 59.86, *SD* = 13.59) (see Table 2).

The tenacity score significantly differed across grades, *F*(3,555) = 4.00, *p* = 0.008 < 0.01, η^2^*_p_* = 0.021; the tenacity score in 10th grade (*M* = 25.79, *SD* = 6.42) was significantly lower than in 11th grade (*M* = 29.15, *SD* = 7.47). The strength score significantly differed across grades, *F*(3,555) = 3.18, *p* = 0.024 < 0.05, η^2^*_p_* = 0.017; the strength score in 10th grade (*M* = 18.76, *SD* = 4.33) was significantly lower than in 11th grade (*M* = 20.64, *SD* = 4.97). The optimism score significantly differed across grades, *F*(3,555) = 3.68, *p* = 0.012 < 0.05, η^2^*_p_* = 0.020; the optimism score in 7th grade (*M* = 9.20, *SD* = 2.82) was significantly lower than in 11th grade (*M* = 10.08, *SD* = 2.37). The optimism score in 10th grade (*M* = 9.15, *SD* = 2.24) was also significantly lower than in 11th grade (*M* = 10.08, *SD* = 2.37) (see Table 2).

**Figure 1:**
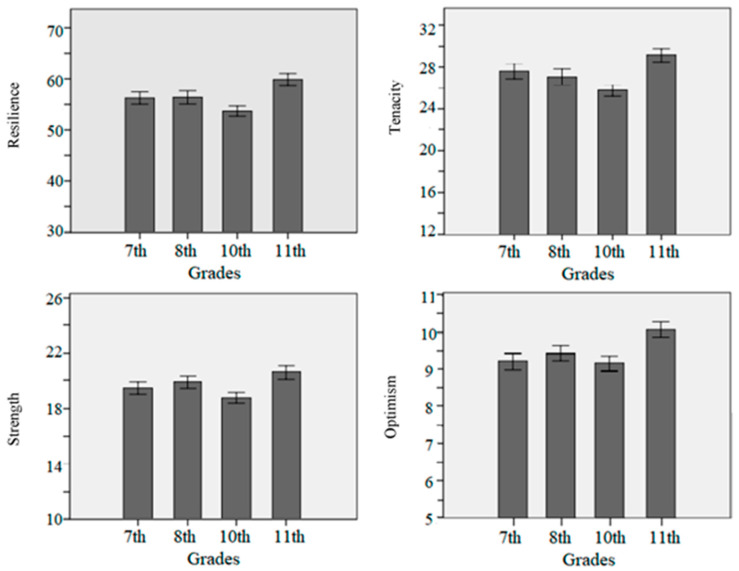
Development pattern of resilience and three factors in all grades. Note. Error bars indicate standard errors.

In Figure 1, it can be found that the score of resilience and three factors (tenacity, strength, and optimism) decreased in the 10th grade and then increased rapidly in the 11th grade, and the score of resilience and three factors (tenacity, strength, and optimism) in the 10th grade and 11th grade reached significant differences.

### 3.2. The Effects of Resilience Interventions for Adolescents of Various Grades

#### 3.2.1. The Effect of Resilience Intervention in the Seventh Grade

**Figure 2:**
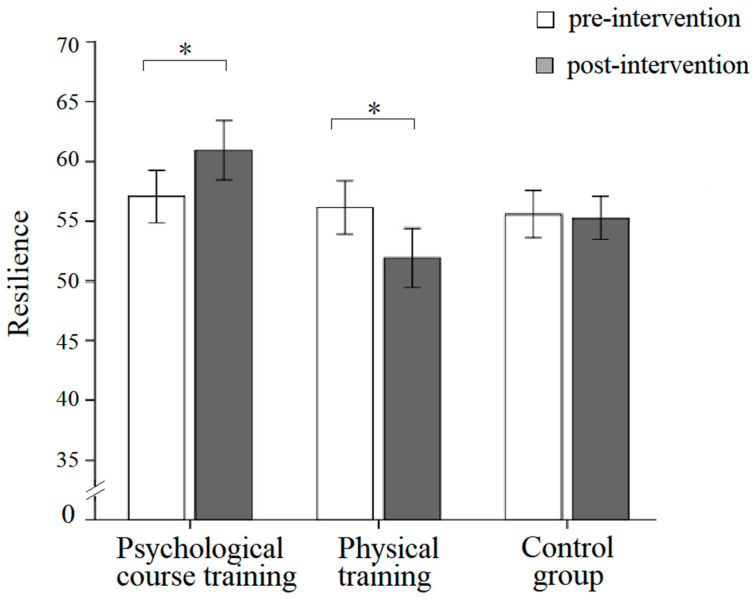
The resilience differences between pre-intervention and post-intervention of three groups in the 7th grade. Note. Error bars indicate standard errors. * *p* > 0.05.

For the resilience score, neither the main effect of the group nor the main effect of time was significant, but the interaction effect between the group and time was significant, *F*(2,144) = 5.17, *p* = 0.007, η^2^*_p_* = 0.067. A simple effect analysis revealed that the resilience score on the post-intervention scale (*M* = 60.94, *SD* = 2.28) was significantly higher than the score on the pre-intervention scale (*M* = 57.04, *SD* = 2.12) for the psychological course training group, *F*(1,144) = 4.79, *p* = 0.03, η^2^*_p_* = 0.03; the resilience score on the post-intervention scale (*M* = 51.92, *SD* = 2.28) was significantly lower than the score on the pre-intervention scale (*M* = 56.12, *SD* = 2.12) for the physical training group, *F*(1,144) = 5.56, *p* = 0.02, η^2^*_p_* = 0.04; there was no significant difference between post and pre-intervention for the control group (see Figure 2).

For tenacity, only the interaction effect was significant, *F*(2,144) = 5.88, *p* = 0.004, η^2^*_p_* = 0.075. The resilience score after intervention (*M* = 30.42, *SD* = 1.26) was significantly higher than the pre-intervention (*M* = 27.92, *SD* = 1.22) for the psychological course training group, *F*(1,144) = 5.32, *p* = 0.02, η^2^*_p_* = 0.04, the resilience score on the post-intervention scale (*M* = 25.72, *SD* = 1.26) was significantly lower than the score on the pre-intervention scale (*M* = 28.18, *SD* = 1.22) for physical training group, *F*(1,144) = 5.15, *p* = 0.03, η^2^*_p_* = 0.04, and there was no time difference for the control group. Neither the main effect nor the interaction term were statistically significant for strength, and optimism.

#### 3.2.2. The Effect of Resilience Intervention in the Eighth Grade

**Figure 3:**
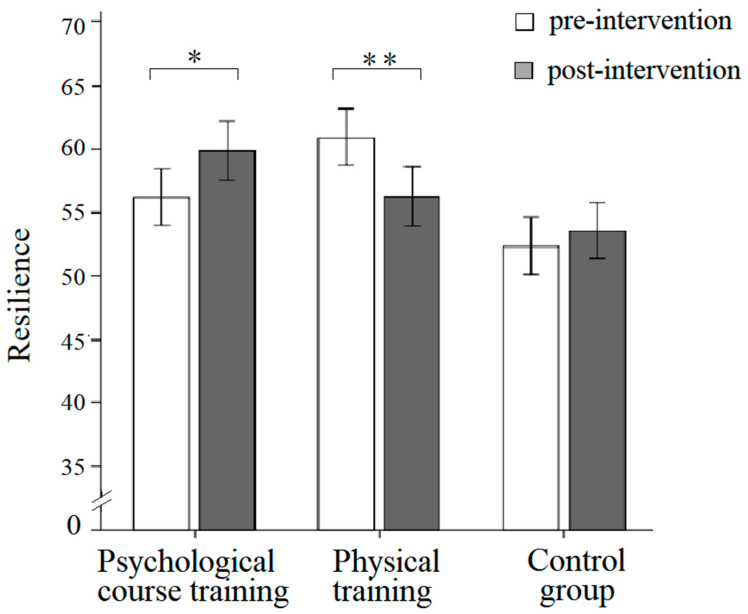
The resilience differences between pre-intervention and post-intervention of three groups in the 8th grade. Note. Error bars indicate standard errors. * *p* > 0.05. ** *p* > 0.01.

For the resilience score, the main effect of group and time was not significant, but there was a significant interaction effect between group and time, *F*(2,145) = 6.83, *p* = 0.001, η^2^*_p_* = 0.086. A simple effect analysis showed that the resilience score after intervention (*M* = 59.85, *SD* = 2.38) was significantly higher than the score in pre-intervention (*M* = 56.17, *SD* =2.32) for the psychological course training group, *F*(1,145) = 4.75, *p* = 0.03, η^2^*_p_* = 0.03. For the physical training group, the resilience score on the post-intervention scale (*M* = 56.24, *SD* = 2.31) was significantly lower than the score on the pre-intervention scale (*M* = 60.96, *SD* = 2.25), *F*(1,145) = 8.33, *p* = 0.004, η^2^*_p_* = 0.05. For the control group, there was no significant difference between post and pre-intervention (see Figure 3).

For tenacity, the main effect was not significant, but the interaction effect showed a difference, *F*(2,145) = 3.80, *p* = 0.03, η^2^*_p_* = 0.050. The tenacity score after intervention (*M* = 27.59, *SD* = 1.28) was significantly lower than the pre-intervention (*M* = 29.65, *SD* = 1.27) for the physical training group, *F*(1,145) = 4.12, *p* = 0.04, η^2^*_p_* = 0.03, while there was no difference for the control group and the psychological course training group. For strength, the interaction term was significant, *F*(2,145) = 10.11, *p* = 0.000, η^2^*_p_* = 0.122, The physical training group got a lower score (*M* = 19.12, *SD* = 0.81) than the pre-intervention (*M* = 21.61, *SD* = 0.80), *F*(1,145) = 16.89, *p* = 0.000, η^2^*_p_* = 0.10. The difference between pre-intervention (*M* = 19.63, *SD* = 0.82) and post-intervention (*M* = 20.85, *SD* = 0.83) for the psychological course training group approached significance, *F*(1,145) = 3.79, *p* = 0.05, η^2^*_p_* = 0.03, while there was no difference for the control group. There was no significant difference in optimism.

#### 3.2.3. The Effect of Resilience Intervention in the 10th Grade

Neither the main effect nor the interaction term was statistically significant for resilience and three factors (*p* > 0.05).

#### 3.2.4. The Effect of Resilience Intervention in the 11th Grade

There was no statistically significant effect on resilience and three factors (*p* > 0.05).

## 4. Discussion

In order to explore the difference between the level of psychological resilience and intervention effect in adolescents and to find the sensitive period for psychological resilience intervention, we conducted a resilience intervention in the four grades. The results showed that resilience remained stable in junior high school students (grades 7 and 8), but decreased in the first year of high school (grade 10) and then increased in the second year of high school (grade 11). Furthermore, the psychological course training was effective in promoting resilience in the 7th and 8th grades, but not in the 10th and 11th grades. That is to say, psychological resilience interventions may produce the best results in the 7th and 8th grades.

### 4.1. The Resilience Development Pattern in Adolescents

There is no significant trend in the development of resilience among junior high school students in the 7th and 8th grades, and a decrease in the level of resilience occurs in the first year of high school (grade 10). Then, there was a significant increase in the level of resilience in the second year of high school (grade 11). This partly confirms hypothesis 1—mental resilience improved only in grade 11, not with each grade. The decrease in the level of resilience in 10th grade (the first year of high school) may be related to the promotion to high school. The study was carried out during the pandemic, and the teenagers in 10th grade not only had to address the challenges in the social milieu caused by the pandemic, but also had to face the changes in the learning circumstance brought about by their physical maturity in puberty [64,65]. They entered new schools and classes, met strange teachers and classmates, and had to acclimatize to the changed conditions. Individuals are prone to fluctuations in resilience when faced with new environments [66,67]. Dual environmental pressure makes the fluctuation more pronounced in the 10th grade. Additionally, 10th-grade students are transitioning to high school and need to prepare for the Gaokao (China’s national higher education entrance examination). The Gaokao is the largest and most important exam for individuals in China [44], and high school students face more fierce competition and tremendous academic pressure than ever before [44]. Meanwhile, students were mandated to study alone at home during the pandemic, lacking face-to-face interaction with teachers and classmates [11,44]. The pandemic changed the form of study, online study predominated, and academic stress increased accordingly [68]. Academic stress is an important factor impacting adolescent resilience development, and excessive academic stress is associated with lower resilience [44].

There was a significant increase in the resilience level among students in grade 11 compared to the first year of high school (grade 10). Research has suggested that older age is associated with higher resilience [66]. Older individuals exhibited higher resilience, especially in regard to emotional regulation and problem solving [61]. The students in 11th grade have long accommodated themselves to the new school and class. Their resilience was less impacted by the changes in study circumstances compared to the students in 10th grade. The increase in higher-order cognitive abilities is characteristic of the transition from adolescence to adulthood and is accompanied by improvements in the structure and function of the brain regions that support them [69]. The component processes that constitute cognitive control continue to mature throughout adolescence and early adulthood. The ability to coordinate these processes to guide thought and action steadily increased during this period [36]. Following exposure to a stressor, a canonical hormonal response mediated by the hypothalamic-pituitary-adrenal (HPA) axis is activated and enables an individual to conquer and adapt to the stress. The axis matures to an adult-like state during pubertal development, with corresponding changes in the activation and recovery of the HPA response [17]. The way of thinking, action, and stress coping continues to develop and becomes close to the level of adulthood in late adolescence [70]. The adolescents in senior class can better adjust and deal with stress maturely and rationally [71]. Taken together, a more stable environment and mature physiological and psychological development may greatly contribute to the higher level of resilience achieved by 11th-grade students.

### 4.2. The Critical Period of Resilience Intervention for Adolescents

The results show that the resilience interventions had a significant influence on teenagers in the 7th and 8th grades, with no significant effect on the 10th and 11th grades. This corroborates hypothesis 2—the intervention is more effective for adolescents in lower grades (grade 7 and grade 8). Based on this, our study found that the sensitive period for resilience intervention was in the 7th and 8th grades.

Adolescence is a critical time for increasing brain plasticity, developing key neurobiological circuits, and sensitivity to the social environment [52,53]. It may also provide a critical opportunity to foster resilience by providing a window for ‘recalibration’ of specific biological systems after adversity [15,52,67]. The intrapersonal factors associated with resilience, such as self-regulation, affective learning, and reward processing, develop rapidly during adolescence [18,53]. Resilience interventions in these factors can reduce the negative impact of stress on individuals. Resilience can manifest even in reduced impact of early adversity on a circuit through neurobehavioral adaptations in neurodevelopment [72].

The imaging studies suggest that adolescence is a period when the need for self-regulation is greatest in a variety of emotional and social settings [45]. Resilience is not merely inertia or insensitivity to stressors, or simply a passive response to adversity, but the result of active, dynamic adaptation [61]. The COVID-19 pandemic has had a profound and devastating impact on the health and development of adolescents worldwide [73]. Still, resilience can help them actively cope with stress and keep them mentally healthy. Our study shows that the resilience intervention, especially the psychological course training, can play a role in significantly improving resilience among 7th and 8th graders. Therefore, providing adolescents with appropriate resilience intervention in the critical period can improve their level of resilience and help them overcome and adapt to stressors actively, deal with adversity better, and ease the suffering of the pandemic.

Students in adolescence are constantly developing, and the resilience interventions delivered at different grades may yield inconsistent results due to the rapid development in physiology and psychology. The factors that promote the development of resilience in adolescence include self-regulation, protective narrative, and cognitive control [15,67,74]. The brain regions involved in emotion regulation, such as the anterior cingulate cortex (ACC) and ventromedial prefrontal cortex (vmPFC) [57], along with the hippocampus associated with protective narratives [36,75] and the prefrontal cortex and parietal cortex related to cognitive control [15,46,53], continue to develop and increasingly integrate throughout adolescence [15,76], and even achieve adult-like levels in many functions [54,55]. There are age-related differences in the prefrontal cortex (PFC), with a central role in resilience. Younger adolescents may not be able to reconcile cognitive control demands with valuable information to adjust behavior strategically [15,74], while older ones are more likely to perform the best strategies to enhance cognitive control [59,77,78]. Hence, high school students may have a high level of resilience owing to the maturity of physiological and psychological foundations, which promote resilience development in late adolescence. General intervention protocols designed to address the process and influencing factors of resilience may have little effect on them. When carrying out resilience intervention for high school students in late adolescence, consideration could be given to developing more specific plans for coping with stressors (e.g., academic pressure, intimate relationships, etc.) for further research.

The study found that four weeks of psychological course intervention can significantly promote the resilience level of junior high school students. The protocol was designed to cover all factors of the resilience scale (i.e., strength, optimism, and tenacity) and had been rehearsed and refined several times. The lessons are rich in content and contain games, activities, stories, and personal sharing that allow students to gain a deeper understanding of the topic. As for the negative effect of physical training, it may be related to the task. The task of hand-muscle development and the spectatorship of classmates and teachers may have created stress for the students. The challenge model posits a curvilinear relation between stress and outcome. Individuals exposed to low and high levels of risk factors are more likely to have negative outcomes, but exposure to moderate levels of adversity is more likely to have positive outcomes [59,75]. Moderate pressure inoculates against subsequent adverse exposures and leads to better health, while excessive or low stress may not trigger the individual coping reaction, and may even have adverse effects [79]. Our study shows that it is feasible and meaningful to carry out mental resilience training in adolescents. It can help teenagers face difficulties better.

### 4.3. Limitations and Future Directions

Some limitations exist in our study. First, the research was not conducted in all grades of both junior high and high schools. Due to the preparation for Zhongkao (entrance examination for a high school) and Gaokao, the students in grade 9 and grade 12 were not investigated and trained. The lack of data in these two grades makes our results less generalizable. Second, training times and duration can be extended. Both the hand-muscle developer and the course training impacted the participants in our study, but longer training times may have led to more stable results, especially for the hand-muscle developer group. Physical training may require longer training duration and more training times to produce stable results. Therefore, future studies may consider extending the training duration to eight weeks and beyond. Third, a variety of physical exercise forms could be tried. Even though the physical training methods used in this study did not improve the level of mental resilience of adolescents, we still believe that physical training could improve the level of resilience [80]. The training intensity and time of the hand-muscle developer were less than other exercise methods. Further studies could try other exercises such as Taekwondo and Tai Chi [49]. Finally, more demographic variables (such as parental economic level, parental status and so on) influencing resilience could be included in the study, with the aim of achieving more accurate findings [81].

## 5. Conclusions

The study measured the psychological resilience of a total of 559 participants in four grades: grade 7 and grade 8 in a junior high school, and grade 10 and grade 11 in a high school. It was found that the resilience level of the adolescents decreased in grade 10 and then increased significantly in grade 11. A four-week resilience intervention was conducted in the four grades using both psychological course training and physical training. The results revealed that the psychological course training was effective in promoting youth resilience, but showed a variation in different grades. This was evidenced by a significant increase in psychological resilience in the 7th and 8th grades, but not in the 10th and 11th grades. The result suggests that the 7th and 8th grades may be a critical period for psychological resilience interventions for adolescents. In addition, our study found some trends in the development of adolescent resilience—mental resilience remained stable in 7th and 8th grade, decreased in 10th grade and increased in 11th grade. Future studies may examine the reasons for the trends to contribute to a better understanding of adolescent development and growth. At the same time, implementing different intervention methods for adolescents at different stages is another direction worthy of attention. We found that normal intervention is effective for sensitive younger adolescents in lower grades, but it may not affect senior teenagers as easily. Therefore, developing intervention methods suitable for the characteristics of adolescents in different grades warrants further study and practice. Based on the results of this study, we look forward to promoting the resilience level of teenagers more effectively and assisting them in coping with the challenges posed by the pandemic.

## Figures and Tables

**Table 1:** The theme and content of the psychological course (developed in relation to the Connor-Davidson resilience scale).

Time	Theme	Sample of Content
The 1st week	Strength	Module 1: Clap hands in 30 s without stopping and experience the unlimited potential you have. Module 2: Self-portrait—express yourself as a plant or an animal. Module 3: Write down three of your strengths, and group members will add more of your advantages. Module 4: Share your most successful experience in class and realize you are great.
The 2nd week	Optimism	Module 1: Read a story of a happy farmer, and learn to be content with what you have. Module 2: Watch a video of dancing during the pandemic, and feel the optimism of medical caregivers and patients. Module 3: Read the fable “a blessing in disguise” and share your ideas. Module 4: Judge events from different perspectives with teachers’ guidance by the ABC Theory of Emotion.
The 3rd week	Tenacity (part 1)	Module 1: Watch Churchill’s speech which said the secret of success is never giving up. Module 2: Fill in the blanks of famous poems which talk about hardiness. Module 3: Feel your own willpower through an arm-raising activity. Module 4: Watch the story of “Nick Vujicic” and acquire his spirit.
The 4th week	Tenacity (part 2)	Module 5: Read the psychologist’s experiment and internalize the importance of setting goals with respect to hardiness. Module 6: Play the game “Who is the Wooden Man”—the people who remain still until the end will be the winners. Module 7: Stand on one foot and keep your body balanced, count how long you persist, then share your feelings.

**Table 2:** Means and standard deviations of resilience and three factors in all grades.

	7th Grade (*n* = 147)	8th Grade (*n* = 148)	10th Grade (*n* = 131)	11th Grade (*n* = 133)
	*M* (*SD*)	*M* (*SD*)	*M* (*SD*)	*M* (*SD*)
Resilience	56.26 (14.90)	56.39 (16.05)	53.69 (11.53)	59.86 (13.59)
Strength	19.44 (5.24)	19.88 (5.70)	18.76 (4.33)	20.64 (4.97)
Optimism	9.20 (2.82)	9.43 (2.76)	9.15 (2.24)	10.08 (2.37)
Tenacity	27.62 (8.61)	27.08 (9.07)	25.79 (6.42)	29.15 (7.47)

## Data Availability

The data of this study are available from the corresponding author upon reasonable request.
